# Data-Driven Multimodal Subtyping Reveals Differential Cognitive Risk and Treatment Effects in the All of Us Cohort

**DOI:** 10.64898/2026.02.10.26345240

**Published:** 2026-02-11

**Authors:** Yinjun Zhao, Karen Marder, Yuanjia Wang

**Affiliations:** 1. Department of Biostatistics, Columbia University Irving Medical Center, New York, NY, USA, 10032; 2. Department of Neurology, Columbia University Irving Medical Center, New York, NY, USA, 10032

**Keywords:** multi-modal integrative subtyping, differential cognitive risk, treatment effect heterogeneity, All of Us Research Program

## Abstract

**INTRODUCTION:**

Cognitively unimpaired (CU) adults show substantial variation in their risk of developing mild cognitive impairment (MCI), yet most subtyping approaches emphasize neurobiological or cognitive features rather than modifiable risk factors.

**METHODS:**

We applied a novel integrative subtyping approach to 121,322 CU adults aged ≥50 years in the All of Us Research Program. Baseline comorbidities, lifestyle behaviors, and social determinants of health were jointly modeled to identify subtypes. Subtype-specific MCI risk was evaluated using Cox model, and medication effects was assessed using double/debiased machine learning model.

**RESULTS:**

Four subtypes with distinct medical, behavioral, and social vulnerability profiles were identified. A cardiometabolic–depressive subtype showed the highest MCI risk and the greatest benefit from cardiometabolic medications, whereas subtypes dominated by behavioral or social vulnerability showed limited benefit.

**DISCUSSION:**

Integrative subtyping based on upstream risk factors reveals differential cognitive risk and treatment response, supporting more targeted and equitable dementia prevention strategies.

## Introduction

Mild cognitive impairment (MCI) is a common intermediate stage between normal aging and dementia, affecting approximately 15–20% of U.S. adults aged 60 years or older^1^. Because disease-modifying therapies for dementia remain limited, with only two monoclonal antibody treatments currently approved and in clinical use after the withdrawal of aducanumab from the market^2^, prevention efforts increasingly focus on identifying cognitively unimpaired (CU) individuals at elevated risk for future cognitive decline. In this context, accurate risk stratification among CU adults is central to targeted prevention, trial enrichment, and efficient allocation of preventive resources. Cognitive impairment and dementia are influenced by a broad range of modifiable risk factors, including cardiometabolic disease, depression, sensory impairment, social isolation, and socioeconomic disadvantage^2,3^. A growing body of data-driven research has demonstrated substantial heterogeneity among cognitively unimpaired (CU) individuals with respect to subsequent risk of mild cognitive impairment (MCI) and dementia. Most existing subtyping studies in CU populations have concentrated on neurobiological and cognitive modalities, including structural magnetic resonance imaging (MRI), such as cortical thickness and regional brain volume; amyloid and tau positron emission tomography (PET); fluid biomarkers, including cerebrospinal fluid and blood-based biomarkers; and detailed neuropsychological profiles^4-7^. Using unsupervised clustering, latent class models, and disease-progression frameworks, these studies have shown that individuals who are clinically normal at baseline may nonetheless occupy distinct biomarker- or cognition-defined subgroups with markedly different longitudinal trajectories and rates of conversion. However, by focusing on downstream pathology, these approaches offer limited insight into upstream, modifiable drivers of risk and have restricted utility for population-level prevention.

In contrast, epidemiologic research has consistently implicated comorbid disease burden, lifestyle behaviors, and social determinants of health (SDOH) as central contributors to cognitive aging. Multimorbidity, particularly cardiometabolic and vascular conditions, has been robustly associated with elevated risk of incident MCI and dementia in large population-based cohorts^8-10^. Lifestyle factors such as smoking, physical inactivity, and obesity further accelerate cognitive decline and account for a substantial proportion of potentially preventable dementia cases^11^. Socioeconomic disadvantage, neighborhood deprivation, and psychosocial stress have likewise been linked to increased dementia incidence and faster cognitive aging^2,12^. Despite this strong evidence base, most studies treat these factors as covariates or examine them in isolation, emphasizing population-average associations and obscuring the co-occurring vulnerability profiles and distinct risk pathways present in real-world populations^2,13,14^. The All of Us Research Program provides a unique opportunity to address this gap. As a national precision-medicine initiative designed to reflect the diversity of the U.S. population, All of Us integrates electronic health records with detailed lifestyle and SDOH survey data, enabling large-scale evaluation of how biological, behavioral, and social factors jointly shape cognitive risk^15^. However, these data are high-dimensional, correlated, and measured on mixed scales, posing challenges for conventional clustering and feature-based multimodal methods that are not designed to jointly model heterogeneous data types.

To overcome these challenges, we apply MINDS, a Bayesian integrative subtyping framework that jointly models binary and continuous variables and identifies latent constructs capturing shared variation across multimodal domains^16^. By integrating comorbidity burden, lifestyle behaviors, and SDOH indicators into a unified latent space, MINDS enables discovery of interpretable subtypes that reflect real-world vulnerability profiles rather than single-modality patterns.

In this study, we leverage All of Us data to: (1) identify multidimensional subtypes among cognitively unimpaired adults using integrative modeling of comorbidities, lifestyle factors, and SDOH; (2) evaluate subtype-specific risk of progression to MCI; and (3) assess whether the effects of antihypertensive and non-GLP antidiabetic and GLP-class therapies on delaying MCI differ across subtypes using a double/debiased machine learning framework. We focused on these medications because hypertension and type 2 diabetes are established upstream cardiometabolic risk factors for cognitive decline and dementia^2,13,17,18^. These therapies have shown potential to delay cognitive impairment in both randomized trials and large meta-analyses^14,19-21^. By explicitly modeling heterogeneity in both baseline risk and treatment responsiveness, this work aims to advance precision prevention for cognitive aging and provide a population-level framework for aligning intervention strategies with dominant vulnerability pathways.

## Methods

### Integrative subtyping framework: MINDS

To identify data-driven subtypes that capture heterogeneity across comorbidities, lifestyle behaviors, and social determinants of health, we employed the Mixed INtegrative Data Subtyping (MINDS) framework, a Bayesian hierarchical model for joint analysis of mixed-type data^16^. MINDS was originally developed to integrate multimodal clinical, cognitive, and neuroimaging data for psychiatric subtyping and is designed to perform simultaneous dimension reduction and clustering while preserving shared structure across heterogeneous modalities.

At its core, MINDS models each data modality through a modality-specific likelihood, i.e., Bernoulli for binary indicators and Gaussian for continuous measures, linked by shared cluster-specific latent centers and subject-specific latent construct. Individuals are assumed to belong to one of the latent clusters, each characterized by distinct mean profiles in the latent construct space, while allowing subject-specific deviations. This formulation enables coherent probabilistic integration of diverse data types without requiring ad hoc transformations or two-stage clustering procedures.

Computationally, MINDS leverages Pólya–Gamma data augmentation to yield conditionally conjugate posterior distributions for binary outcomes, enabling efficient Gibbs sampling for posterior inference^22^. The number of subtypes is selected using a Bayesian information criterion that balances model fit and parsimony, as described in the original methodological work^16^.

### Cohort for subtyping

We applied the MINDS framework to identify data-driven subtypes among participants in the All*of*Us Research Program by jointly integrating comorbidities, social determinants of health (SDOH), and lifestyle factors. The Curated Data Repository (CDR) Version 8, released by the All*of*Us Research Program and containing participant data through October 1, 2023, served as the source for all analyses^23^.

**Figure 1:**
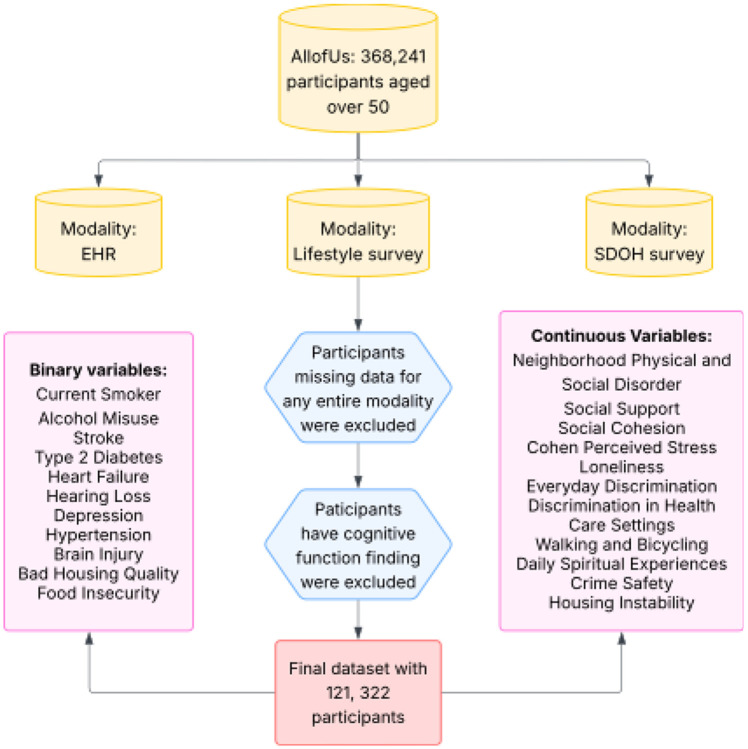
Flowchart of participant selection and exclusion criteria in the All of Us cohort. Participants missing data for any entire modality were excluded from the analysis.

We began with all eligible participants aged 50 years or older and linked their electronic health records (EHR), SDOH survey responses, and lifestyle survey data. Participants missing an entire modality at baseline or with documented cognitive function finding at baseline were excluded to reduce potential bias arising from systematically different health or survey-response profiles. Participants with any recorded cognitive function finding were defined as the SNOMED CT parent concept “Cognitive function finding” (OMOP concept id 4162723) and all its descendant concepts, identified in the OMOP CONDITION domain. This definition captures clinician-documented cognitive abnormalities (e.g., memory, attention, or thought findings). The final analytic cohort consisted of 121,322 individuals with complete multimodal data and follow-ups to assess incident MCI. Figure 1depicts the derivation of this cohort.

Comorbidity status at baseline including stroke, type 2 diabetes, heart failure, hearing loss, depression, hypertension, and brain injury was obtained from EHR data. Lifestyle behaviors were extracted from the lifestyle survey. Among current drinkers, we constructed the AUDIT–C score (range, 0–12) using three items assessing (1) drinking frequency, (2) typical quantity consumed per drinking occasion, and (3) frequency of binge drinking (defined as consuming 6 or more drinks on a single occasion). Each item included five ordered response options scored from 0 (lower consumption) to 4 (higher consumption). Alcohol misuse was defined as an AUDIT–C score of 3 or higher for women and 4 or higher for men^24^. Current smoker status was obtained from the corresponding lifestyle survey question.

SDOH measures were obtained from validated instruments included in the SDOH survey. These measures encompassed four conceptual domains: social and community context, economic stability, neighborhood and built environment, and health and healthcare^25^. Constructs such as social cohesion, social support, loneliness, perceived discrimination, perceived stress, and daily spiritual experiences were assessed through established multi-item scales. Economic stability was evaluated using indicators of food insecurity, housing instability, and housing quality. Neighborhood characteristics, including physical and social disorder, walk ability, and crime, were captured using standardized environmental assessment tools, and perceived discrimination in healthcare settings was assessed using a dedicated subscale. Higher values of the SDOH measures reflected worse situation, consistent with the direction of comorbidity and lifestyle measures. Detailed descriptions of each construct and measurement instrument are provided in Table A. 2. Collectively, these multidimensional measures served as inputs for the subtyping analysis.

### MCI association analysis

To investigate the prognostic relevance of the identified subtypes, we conducted an exploratory survival analysis to examine the association between subtypes and the subsequent risk of developing mild cognitive impairment (MCI). Time-to-event was defined as the interval from cohort entry to the first documented diagnosis of MCI or censoring, whichever occurred earlier. Kaplan–Meier curves were generated to provide an initial descriptive comparison of incident MCI across clusters. We then fit Cox proportional hazards models to estimate hazard ratios (HRs) and 95% confidence intervals (CIs) for MCI associated with each subtype, controlling for demographics and antihypertensive, non-GLP antidiabetic and GLP class medication use at baseline. The medication use was ascertained from electronic health records based on prescriptions recorded within one year before the baseline assessment. Participants with evidence of use of any medication in a given class during this one-year window before baseline were classified as users of that medication class. Details of the agents that were included in the medication class are in Table A. 3.

### Treatment causal effect analysis

We estimated the causal effects of antihypertensives, non–GLP antidiabetic, and GLP-class medications use on time to incident MCI using a double/debiased machine learning interactive regression model (DoubleML-IRM)^26^. Medication use was treated as a binary exposure, and confounding variables included age, sex, race/ethnicity, educational attainment, and baseline cardiometabolic comorbidities.

The primary estimand was the average treatment effect (ATE), defined as the expected difference in outcomes had all individuals received versus not received the treatment. To assess heterogeneity in treatment response, we additionally estimated group average treatment effects (GATEs), which quantify treatment effects within each data-driven subtype identified by the integrative subtyping model. The outcome was defined as the logarithm of observed follow-up time to MCI, such that treatment effects can be interpreted on a multiplicative time scale. Exponentiating the estimated effects yields a geometric mean time ratio, representing the relative prolongation (or shortening) of MCI-free survival associated with medication use.

To account for right censoring, we incorporated inverse probability of censoring weights (IPCW). Specifically, we estimated the probability of remaining uncensored over time conditional on treatment status and covariates, and weighted complete observations by the inverse of this probability. This procedure reduces bias due to informative censoring under standard assumptions^27^. A fixed cutoff time of 48 months was used to define the at-risk set, including individuals who either experienced the event before this time or remained under observation beyond it.

## Results

### Subtyping results

We evaluated models with the number of clusters ranging from k=3 to k=9 using the information criterion^28^ to determine the optimal cluster structure. As shown in Figure A. 1(a), the IC reached its minimum at k=4, indicating that the four-cluster model achieved the best balance between model fit and parsimony. To assess convergence and model stability, we examined posterior log-likelihood trace plot in Figure A. 1(b), which demonstrated stable convergence and consistent mixing of the Markov chain Monte Carlo (MCMC) samples after the burn-in period. These results suggest that the four-cluster solution provides a well-fitted and robust model for integrating the multimodal data.

As shown in Figure 1, the four subtypes demonstrated distinct and coherent patterns across comorbidity burden, lifestyle risk behaviors, and social determinants of health. Subtype I reflected a broadly healthy profile, characterized by minimal chronic disease, low rates of smoking and alcohol misuse, and consistently favorable social and environmental conditions, suggesting a Low-Risk Healthy Aging group. Subtype II showed markedly elevated smoking and alcohol misuse alongside the highest levels of social and economic adversity, including neighborhood disorder, discrimination, social isolation, and housing instability, indicating a Behavioral-Risk and Socially Vulnerable subgroup. Subtype III exhibited the greatest medical burden, with pronounced hypertension, depression, hearing loss, and cardiometabolic disease, paired with moderate psychosocial stressors, aligning with a Cardiometabolic–Depressive Multimorbidity subtype. Finally, Subtype IV demonstrated intermediate levels of comorbidity and moderately elevated SDOH disadvantage, representing a Mixed-Risk Mild Vulnerability group. Together, these patterns highlight the multidimensional heterogeneity in health, lifestyle, and social context within the population. The characteristics of the four types are summarized in Table 1, which are also reflected in the summary statistics by the four subtypes in Table A. 1.

**Figure 3:**
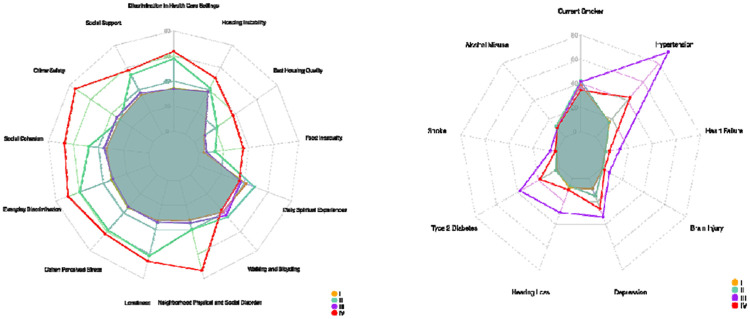
Radar plot illustrating differences in SDOH and comorbidity profiles across the four identified latent subtypes (I–IV). Each spoke represents a standardized measure. Larger polygon areas indicate higher standardized scores for a given subtype. The figure highlights distinctive SDOH and comorbidity burden patterns across subtypes.

**Figure 4:**
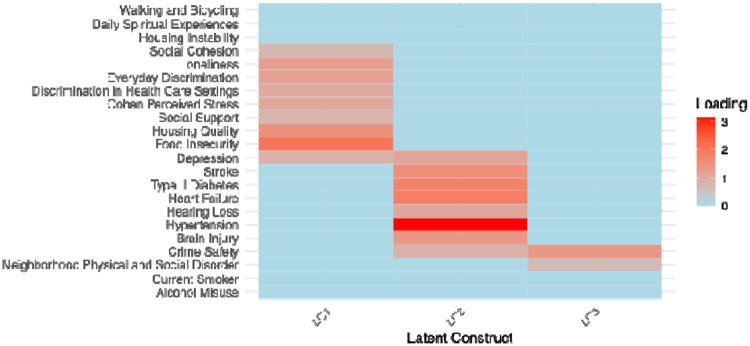
Heatmap displaying the magnitude of standardized loadings for each observed measure across the three latent constructs (LC1–LC3). Measures include clinical, lifestyle indicators, and social determinants of health. Absolute loadings smaller than 0.6 were thresholded to zero to emphasize only the most influential measures. Darker colors indicate stronger loadings, highlighting the differential contribution of each measure to the three latent constructs.

The radar plots provide a visual comparison of subtype differences across SDOH, lifestyle, and comorbidity measures in another way. The first plot (Figure 3) illustrates variation in social determinants of health across the four subtypes, highlighting pronounced contrasts in constructs such as discrimination in healthcare, social support, social cohesion, perceived stress, loneliness, neighborhood disorder, and material hardship (e.g., food insecurity, housing instability). Subtypes II and IV show elevated levels on multiple adverse SDOH indicators, whereas Subtype I generally reflects more favorable social and environmental conditions. The second plot (Figure 3) summarizes subtype differences in lifestyle risk factors and chronic disease burden, including smoking, alcohol misuse, and major comorbidities such as hypertension, diabetes, heart failure, stroke, and depression. Subtype III shows elevated levels on comorbidities. Together, these plots highlight distinct multidimensional profiles across subtypes, demonstrating how combinations of social context, behavior, and health conditions jointly characterize the heterogeneity captured by the clustering model. The three latent constructs that MINDS estimated reflect distinct but complementary domains of vulnerability. As shown in Figure 4, LC1 is best characterized as Neighborhood Disorder and Safety Risk, defined by high levels of neighborhood physical and social disorder and low perceptions of crime safety, indicating exposure to adverse environmental conditions. LC2 represents a pattern of High-Burden Chronic Multimorbidity, marked by strong associations with stroke, type 2 diabetes, heart failure, hypertension, hearing loss, brain injury, and depression. LC3 reflects Psychosocial Stress and Social Vulnerability, driven by elevated food insecurity, poor housing quality, loneliness, discrimination (every day and within health-care settings), perceived stress, and low social support and cohesion. See Table A. 4 for the exact values of the loading matrix.

Figure A. 1 illustrates the distribution of the three latent constructs (LC1–LC3) across the four subtypes, highlighting clear and interpretable subtype-specific patterns. Subtype I shows consistently low to moderate expression of LC1 and minimal contributions from LC2 and LC3, reflecting a comparatively low burden of adverse social and health factors. Subtype II demonstrates a stronger LC1 profile, driven by elevated social and psychological stressors such as loneliness, discrimination, and perceived stress. In contrast, Subtype III is distinguished by a dominant LC2 pattern, with high scores on multiple comorbidities including hypertension, type 2 diabetes, hearing loss, depression, and other chronic conditions. Subtype IV exhibits the strongest expression of LC3, characterized by markedly higher levels of neighborhood physical/social disorder and crime safety concerns. Together, these distinct profiles demonstrate that the subtypes are differentiated not only in magnitude but also in the composition of their latent constructs, capturing qualitatively different combinations of social, environmental, and health-related vulnerabilities.

### Subtype’s association with MCI

The models were first examined unadjusted (Table A. 5) and then adjusted for relevant demographic, clinical, and medication-use covariates(Table 2) to explore whether the observed associations persisted after accounting for potential confounders.

**Figure 5:**
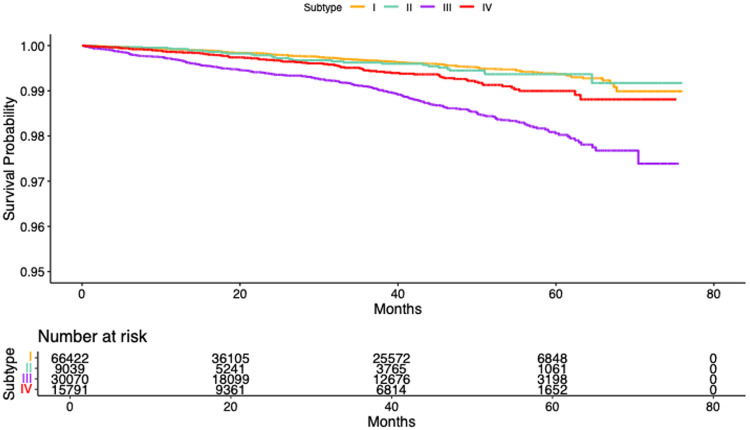
Kaplan–Meier survival curves depicting the probability of remaining free from MCI across Subtypes I–IV. The curves illustrate clear separation between groups, with Subtype III exhibiting the most rapid decline in survival probability, followed by Subtypes IV and II, while Subtype I shows the highest MCI-free survival over time. The number at risk at each time point is shown below the plot.

The four data-driven subtypes demonstrated clear heterogeneity in their risk of developing mild cognitive impairment (MCI). Using Subtype I as the reference, individuals in Subtype III exhibited the highest risk, with a hazard ratio (HR) of 3.69 (95% CI: 3.14–4.33), indicating more than a threefold increase in the hazard of MCI. Subtype IV also showed significantly elevated risk (HR = 1.87, 95% CI: 1.48–2.35), whereas Subtype II demonstrated a more modest but still significant increase (HR = 1.43, 95% CI: 1.07–1.91). These subtype differences were further reflected in the Kaplan–Meier survival curves in Figure 5, where Subtype III displayed the steepest decline in MCI-free survival, followed by Subtype IV and Subtype II, while Subtype I maintained the most favorable survival trajectory. Together, these findings indicate that Subtype III represents a particularly vulnerable group with accelerated cognitive decline, whereas Subtype I appears comparatively resilient, with Subtypes II and IV occupying intermediate risk profiles.

### Causal treatment effect

**Figure 6:**
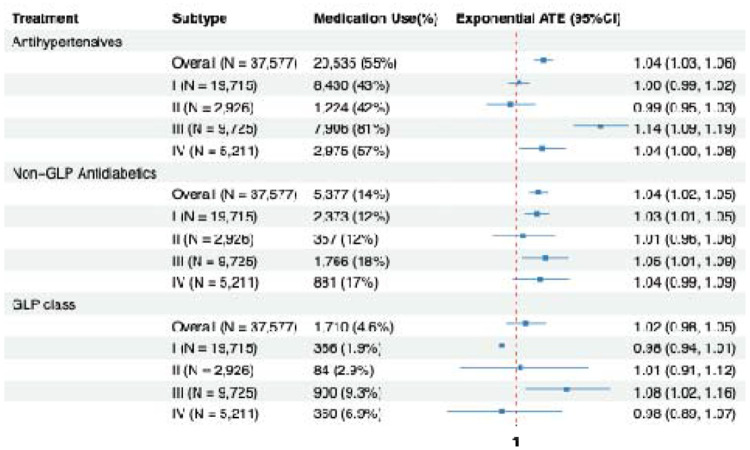
Estimated exponential average treatment effects for antihypertensive, non-GLP antidiabetic and GLP class medications across the four data-driven subtypes. For each subtype and the overall sample, the number of individuals taking each medication class and the corresponding exponential ATE with 95% confidence interval are shown.

Figure 6 displays the estimated exponential average treatment effects (ATEs) of antihypertensive, non–GLP antidiabetic, and GLP-class medications on time to incident MCI in the overall cohort and across four data-driven subtypes. Antihypertensive use led a modestly longer time to MCI overall (time ratio = 1.04; 95% CI, 1.03–1.06), corresponding to an approximately 4% increase in geometric-mean follow-up time(months). Subtype III showed a markedly stronger association (time ratio = 1.14; 95% CI, 1.09–1.19; ~14% longer time), while Subtype IV demonstrated a smaller but statistically significant association (time ratio = 1.04; 95% CI, 1.00–1.08). No significant associations were observed for Subtypes I or II.

For non–GLP antidiabetic medications, the overall association was similarly modest but statistically significant (time ratio = 1.04; 95% CI, 1.02–1.05; ~4% longer time). At the subtype level, significant associations were observed in Subtype I (time ratio = 1.03; 95% CI, 1.01–1.05; ~3% longer time) and Subtype III (time ratio = 1.05; 95% CI, 1.01–1.09; ~5% longer time), whereas Subtypes II and IV showed no evidence of benefit. GLP-class medications were not associated with time to MCI overall; however, Subtype III demonstrated a significant association (time ratio = 1.08; 95% CI, 1.02–1.16; ~8% longer time). Overall, these results indicate pronounced heterogeneity in medication-associated delay of MCI onset, with Subtype III consistently exhibiting the largest estimated benefit across medication classes.

## Discussion

In this large and diverse cohort from the All of Us Research Program, we identified four data-driven subtypes of cognitively unimpaired adults using integrative modeling of comorbidities, lifestyle behaviors, and SDOH. These subtypes captured distinct multidimensional vulnerability profiles that were associated with heterogeneous risk of incident MCI and have differential estimated effect of antihypertensive and antidiabetic medication use. By integrating correlated biological, behavioral, and social risk factors into coherent latent profiles, this study provides a population-level framework for understanding heterogeneity in cognitive aging and offers insight into why cardiometabolic interventions have yielded modest and inconsistent average effects in prior dementia-prevention studies^2,10,29^.

The four identified subtypes reflected distinct configurations of medical burden, behavioral risk, and social disadvantage. Subtype I represented a low-risk healthy aging profile, characterized by minimal chronic disease, low smoking and alcohol misuse, and favorable social conditions. Subtype II was marked by prominent behavioral risk and psychosocial adversity, but relatively limited cardiometabolic burden. Subtype III exhibited concentrated cardiometabolic and depressive multimorbidity, particularly hypertension, diabetes, hearing loss, and depression, with comparatively less social disadvantage. Subtype IV combined moderate medical comorbidity with pronounced social and environmental adversity, including neighborhood disorder, food insecurity, and housing instability.

These patterns align with prior epidemiologic studies of comorbidity and social disadvantage associated with adverse cognitive outcomes^8,12,30^. Unlike studies that model comorbidities or SDOH as covariates or examine single domains in isolation^2,31-33^ our integrative approach treats these factors as defining dimensions of heterogeneity, yielding subtypes that more closely reflect real-world co-occurring risk profiles.

Consistent with epidemiologic evidence, the subtypes showed marked differences in risk of incident MCI. Subtype III exhibited the highest hazard, aligning with evidence that cardiometabolic and vascular disease burden strongly predicts cognitive impairment and dementia^8,32,34^. Subtype IV also had substantially elevated risk, supporting links between chronic social and environmental disadvantage and accelerated cognitive aging ^2,33,35^. In contrast, Subtype II showed a more modest increase in risk, while Subtype I maintained the most favorable MCI-free survival trajectory.

Notably, the relatively modest MCI risk observed in Subtype II, despite substantial behavioral and psychosocial adversity, may reflect delayed manifestation of stress-related effects, unmeasured intermediate conditions, or attenuation due to competing risks, differential healthcare engagement, or under-ascertainment of cognitive diagnoses^12,30,35^.

We observed substantial heterogeneity in the estimated effect of cardiometabolic medication use. Subtype III consistently exhibited the largest estimated effect by antihypertensive, non–GLP antidiabetic, and GLP-class medications, suggesting that pharmacologic modification of cardiometabolic risk factors may be most impactful among individuals with substantial underlying comorbidity burden. This pattern is concordant with randomized evidence demonstrating that intensive blood pressure control reduces incident MCI among individuals at elevated vascular risk^14^ and metformin improves memory performance^36^. In contrast, Subtypes II and IV showed limited or selective effects by pharmacologic therapy, suggesting that when cognitive risk is driven primarily by behavioral or structural adversity, medication alone may be insufficient to meaningfully alter trajectories. Subtype I showed modest estimated effects by non–GLP antidiabetic medications despite low baseline MCI risk, raising the possibility that some cardiometabolic therapies may influence cognitive aging through mechanisms beyond prevention of vascular events, such as effects on insulin signaling, inflammation, or cerebral metabolism^21,37^. This interpretation aligns with metformin clinical trials in cognitive aging, including Metformin in Mild Cognitive Impairment and TAME, which target insulin resistance and inflammation as upstream mechanisms of neurodegeneration ^36,38^.

Importantly, these findings should be interpreted as estimated causal effects carefully. Although we applied a double/debiased machine learning causal framework with inverse probability of censoring weights to mitigate confounding and selection bias, residual confounding related to disease severity, medication indication, adherence, healthcare access, and treatment duration cannot be excluded. Failure to account for underlying vulnerability profiles may contribute to the modest and inconsistent average effects reported in prior observational and interventional studies^10,21^.

The latent constructs identified by the integrative model highlight distinct yet interacting pathways linking subtype profiles to cognitive risk and inform targeted prevention strategies. The construct dominated by cardiometabolic and depressive multimorbidity likely reflects vascular, metabolic, and inflammatory mechanisms contributing to neurodegeneration^34^, suggesting that individuals in Subtype III may benefit most from intensified cardiometabolic and neurological disease management. In contrast, the construct characterized by psychosocial stress, discrimination, loneliness, and material hardship may act through chronic stress–related pathways, including hypothalamic–pituitary–adrenal axis dysregulation and reduced cognitive reserve^2^, indicating that Subtype II may respond best to psychosocial and community-based interventions. The construct reflecting neighborhood disorder and safety concerns likely affects cognition indirectly by limiting physical activity, social engagement, and access to health-promoting resources^35^, underscoring the need for structural interventions in Subtype IV. These pathways likely interact over the life course, reinforcing the value of multidimensional risk stratification for mechanism-informed and targeted dementia prevention.

Together, these findings underscore that dementia prevention strategies should be aligned with dominant vulnerability pathways rather than applied uniformly across heterogeneous populations. Integrative subtyping frameworks embedded within large-scale health systems can identify individuals most likely to benefit from pharmacologic modification of upstream cardiometabolic risk factors, while distinguishing subgroups for whom behavioral or structural interventions addressing psychosocial and environmental risk may be more relevant. By explicitly modeling heterogeneity in both baseline vulnerability and treatment responsiveness, this approach supports subgroup-enriched pragmatic trials and advances a more mechanism-informed, targeted, and equitable framework for precision prevention in cognitive aging.

Importantly, this population-level perspective is complementary to biomarker-based approaches. Whereas biomarkers capture downstream, disease-specific biological processes, integrative subtyping focuses on upstream vulnerability relevant to early risk stratification and prevention, together providing a more comprehensive foundation for dementia prevention across the life course^2^.

Several limitations warrant consideration. First, the analysis focused on all-cause MCI, and information on etiologic MCI subtypes and severity was unavailable, as standardized cognitive test scores or continuous measures of cognitive performance were not incorporated. Second, cognitive, neuroimaging, and molecular biomarkers were not included; accordingly, the identified subtypes should be interpreted as population-level vulnerability profiles rather than direct proxies for underlying neuropathology^7^. Third, medication exposure was defined using baseline prescription records and did not capture dose, adherence, or changes over follow-up. Fourth, subtypes were derived from baseline measures and therefore do not capture longitudinal changes that may influence MCI risk or treatment responsiveness. Also, although the All of Us cohort enhances generalizability through its size and diversity^15^, healthy volunteer bias, differential healthcare access, and diagnostic ascertainment may affect absolute risk estimates. Last, subtype definitions are model-dependent, and alternative modeling choices or the inclusion of additional modalities could yield different subgroup structures. Independent validation in external cohorts such as the UK Biobank is therefore needed to assess the robustness, reproducibility, and generalizability of the identified subtypes.

## Conclusion

In summary, this study demonstrates that integrative, data-driven subtyping based on comorbidities, lifestyle behaviors, and social determinants of health can uncover clinically and socially meaningful heterogeneity in cognitive aging trajectories. The identified subtypes differed substantially in risk of incident MCI and in estimated associations with cardiometabolic therapies, highlighting the importance of aligning prevention strategies with dominant vulnerability pathways. By explicitly recognizing that cognitive risk emerges from interacting biological mechanisms, modifiable health behaviors, and structural determinants, this multidimensional framework provides a foundation for more targeted, equitable, and effective approaches to dementia prevention.

## Figures and Tables

**Figure 2:**
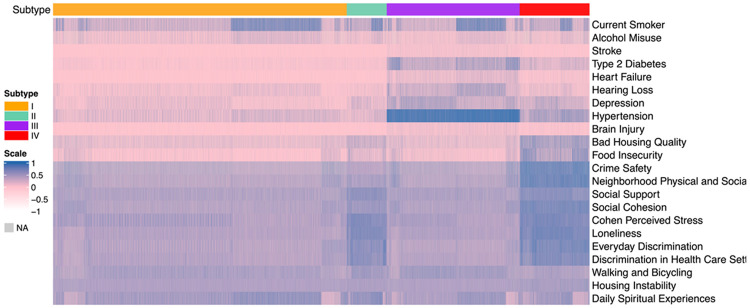
Heatmaps of binary and continuous measures across subtypes by the MINDS method, integrating lifestyle factors, comorbidity and social determinants of health measures. The top heatmap includes two binary SDOH measures, two binary lifestyle risk factors, and seven binary comobidity diagnosis indicators. Bottom heatmap includes eleven SODH measures; scores are normalized by row.

**Table 1:** Characteristics of the four data-driven subtypes based on comorbidity, lifestyle behaviors, and social determinants of health.

Subtype	Key Features	Name
**I**	Lowest comorbidity burden; low smoking and alcohol misuse; favorable social and environmental conditions.	Low-Risk Healthy Aging
**II**	Intermediate chronic disease burden; moderate smoking/alcohol levels; moderate disadvantage across social determinants of health.	Mixed-Risk Mild Vulnerability
**III**	Greatest medical burden with pronounced hypertension, depression, hearing loss, and cardiometabolic disease; moderate psychosocial stress levels.	Cardiometabolic–Depressive Multimorbidity
**IV**	Highest social and economic adversity including neighborhood disorder, discrimination, social isolation, and housing instability; moderate chronic disease.	Social Vulnerability

**Table 2:** Cox Regression Results on time to MCI by subtype, demographics, and medication use.

Characteristic	N	HR	95% CI	p-value
**Subtype**				**<0.001**
I	66,422	—	—	
II	9,039	1.11	0.53, 2.32	
III	30,070	4.71	3.40, 6.52	
IV	15,791	1.96	1.17, 3.30	
**Age (years)**	121,322	1.85	1.69, 2.03	**<0.001**
**Gender**				**<0.001**
Female	74,270	—	—	
Male	47,052	1.37	1.16, 1.61	
**Race**				0.9
Non-White	15,914	—	—	
White	105,408	1.02	0.77, 1.35	
**Ethnicity**				0.2
Non-Hispanic	110,172	—	—	
Hispanic	11,150	1.22	0.88, 1.69	
**Educational attainment**				0.5
Below college	46,794	—	—	
College & above	74,528	0.94	0.79, 1.12	
**Antihypertensive use**				**<0.001**
No	80,649	—	—	
Yes	40,673	3.23	2.35, 4.42	
**Non-GLP antidiabetic use**				0.5
No	108,002	—	—	
Yes	13,320	1.13	0.77, 1.65	
**GLP-1 class use**				0.077
No	116,665	—	—	
Yes	4,657	2.16	1.01, 4.66	
**Subtype × Antihypertensive use**	121,322			**<0.001**
II * Yes	2,605	1.25	0.51, 3.07	
III * Yes	14,025	0.31	0.21, 0.46	
IV * Yes	5,713	0.87	0.47, 1.62	
**Subtype × Non-GLP antidiabetic use**	121,322			>0.9
II * Yes	842	1.19	0.45, 3.10	
III * Yes	4,576	0.94	0.57, 1.54	
IV * Yes	2,071	1.13	0.58, 2.18	
**Subtype × GLP class use**	121,322			0.7
II * Yes	239	0.44	0.05, 3.79	
III * Yes	2,418	0.71	0.30, 1.67	
IV * Yes	928	0.58	0.19, 1.72	

Abbreviations: CI = Confidence Interval, HR = Hazard Ratio

**Figure A. 1:**
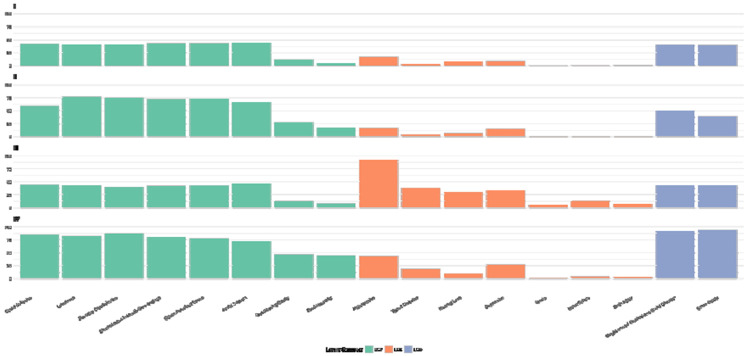
Comparison of comorbidity and SDOH profiles across the four subtypes (I–IV) and three latent constructs. Each panel displays the standardized loadings for a set of clinical comorbidities and SDOH-related measures. The figure highlights the distinct pattern of comorbidity versus SDOH burdens across subtypes and latent constructs.

**Figure A. 2:**
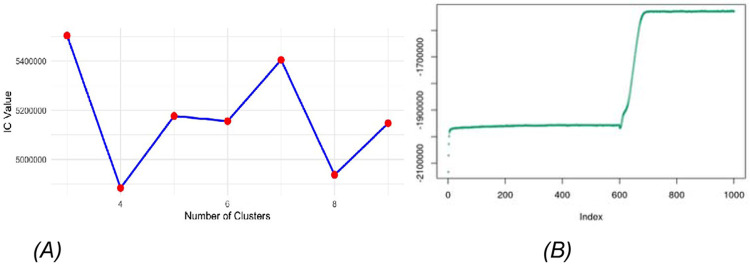
Model selection and posterior log-likelihood trace plots across modalities. (A) Information criterion (IC) values are shown for candidate numbers of clusters ( ), with the optimal model selected at . (B) Posterior log-likelihood trace plots demonstrate good convergence of the Markov chain Monte Carlo (MCMC) sampler when .

## Research in Context

Previous research shows that cognitively unimpaired adults differ widely in their risk of developing cognitive impairment. Most prior subtyping studies have focused on brain imaging or cognitive test results. In contrast, large epidemiologic studies indicate that medical conditions, health behaviors, and social and environmental factors play a major role in dementia risk, but these factors are rarely integrated into data-driven subtyping approaches.

In a large, diverse U.S. cohort, we identified distinct subtypes of cognitively unimpaired adults defined by different combinations of medical, behavioral, and social risk factors. These subtypes differed substantially in their risk of developing mild cognitive impairment and in their response to commonly used cardiometabolic medications. This approach reveals risk patterns not captured by biomarker- or cognition-based subtyping. Integrating medical, behavioral, and social risk factors may improve early identification of individuals at higher risk for cognitive decline and help tailor prevention strategies. Future studies should validate these subtypes in other populations and examine how they evolve over time and interact with biological markers.

## Appendix

### A. Formulation of Integrative subtyping framework: MINDS

The model that integrates binary and continuous modalities is proposed as follows

$$Y_{i\cdot }^{\left(1\right)}|X,b_{i},V,Z_{i},a^{\left(1\right)}∼\text{Bernoulli}\left(\frac{e^{(Z_{i}^{T}X+b_{i}^{T})V-a^{\left(1\right)}}}{1+e^{(Z_{i}^{T}X+b_{i}^{T})V-a^{\left(1\right)}}}\right),$$

$$Y_{i\cdot }^{\left(2\right)}|X,b_{i},U,Z_{i},a^{\left(2\right)},\Sigma ∼\text{Normal}\left((Z_{i}^{T}X+b_{i}^{T})U-a^{\left(2\right)},\Sigma \right),$$

where X denotes the cluster centers shared across modalities, $Z_{i}$ is the cluster membership of subject i, and $b_{i}\in R^{N_{t}}$ represents subject-specific latent constructs. $V\in R^{N_{t}\times N_{d_{1}}}$ and $U\in R^{N_{t}\times N_{d_{2}}}$ are loading matrices for the binary and continuous modalities, respectively, with $N_{d_{1}}$ binary items and $N_{d_{2}}$ continuous measures. The vectors $a^{\left(1\right)}$ and $a^{\left(2\right)}$ denote item difficulty parameters. For the continuous modality, $\Sigma =\text{diag}(\sigma_{1}^{2},\ldots ,\sigma_{N_{d_{2}}}^{2})$.

At its core, MINDS models each data modality through a modality-specific likelihood, i.e., Bernoulli for binary indicators and Gaussian for continuous measures, linked by shared cluster-specific latent centers X and subject-specific latent construct $b_{i}$. Individuals are assumed to belong to one of K latent cluster, each characterized by distinct mean profiles in the latent construct space, while allowing subject-specific deviations.

## B. Treatment causal effect analysis

We use double/debiased machine learning Interactive regression (IRM) model (DoubleMLIRM)(Chernozhukov et al. 2018) to evaluate the causal treatment effect of antidiabetics and antihypertensive medication on MCI. The model takes the form

$$Y=g_{0}(W,X)+\epsilon ,\;E(\epsilon \;|\;W,X)=0,$$

$$E(D)=m_{0}(X),$$

where the treatment variable is binary, W∈{0,1}, Y is the outcome, and X includes other confounding variables. Target parameters of interest in this model are the average treatment effect (ATE), $\theta_{0}=E[Y(1,X)-Y(0,X)]$. Group Average Treatment Effects (GATEs) for DoubleMLIRM consider the target parameters $\theta_{0,k}=E[Y(1)-Y(0)\;|\;G_{k}],k=1,\ldots ,K$, where $G_{k}$ denotes a group indicator and Y(d) the potential outcome with d∈{0,1}. In our analysis, the outcome is defined as the logarithm of the observed follow-up time, Y=logU. Accordingly, the average treatment effect (ATE) is E[logU(1)−logU(0)], and exponentiating this quantity yields the corresponding geometric mean time ratio,

$$\text{exp(ATE)}=\frac{\text{exp}(E[\log U(1)])}{\text{exp}(E[\log U(0)])}.$$

We incorporate weights into the DoubleMLIRM framework using an inverse probability of censoring weighting (IPCW) approach, which has been shown to effectively reduce bias due to censoring under broad conditions (Robins and Finkelstein 2000). Let $T_{i}$ denote subject i’s survival time, $C_{i}$ the censoring time, and $U_{i}=\text{min}(T_{i},C_{i})$ the observed follow-up time. The event indicator is defined as $\Delta_{i}=1\{T_{i}\leq C_{i}\}$. The complete-case at-risk set at a fixed time $t_{0}$ is defined as $1_{\text{comp}}=\{i\in I:\Delta_{i}=1\;\text{or}\;U_{i}>t_{0}\}$, representing individuals who either experienced the event before $t_{0}$ or remained under observation beyond $t_{0}$.

The first step of IPCW is to estimate the conditional survival function,

$$S^{C}(s\;|\;x,w)=P(C_{i}\geq s\;|\;W_{i}=w,X_{i}=x).$$

Estimation is performed using only complete cases. Given an estimator ${\hat{S}}^{C}(\cdot )$, the second step of IPCW assigns a weight to each complete observation,

$${\hat{K}}_{i}=\frac{1}{{\hat{S}}^{C}(\text{min}\{U_{i},t_{0}\}\;|\;X_{i},W_{i})}.$$

In our analysis, the cutoff time was set to $t_{0}=48$ months, confounding variables included age, gender, race/ethnicity, education, comorbidity of diabetes and hypertension. We consider anti-diabetic and anti-hypertensive medication use to be the treatment variables which were defined consistently with the criteria outlined in Section 2.3.

## C. Tables and figures

**Table A. 1:** Summary Statistics by Four Subtypes, n(%) for binary variables and mean(sd) for continuous variables.

Characteristic	I (N=66,422)	II (N=9,039)	III (N=30,070)	IV (N=15,791)
**Lifestyle**				
Current Smoker	25,222 (39%)	3,777 (42%)	12,198 (41%)	5,262 (34%)
Alcohol Misuse	7,217 (11%)	1,007 (12%)	2,829 (9%)	1,437 (10%)
**Comorbidity**				
Stroke	262 (0.4%)	10 (0.1%)	88 (0.3%)	28 (0.2%)
Type 2 Diabetes	1,922 (2.9%)	332 (3.7%)	1,277 (4.3%)	1,023 (6.5%)
Heart Failure	345 (0.5%)	67 (0.7%)	338 (1.3%)	190 (1.2%)
Hearing Loss	5,173 (7.8%)	565 (6.3%)	2,130 (7.1%)	1,058 (6.7%)
Depression	5,763 (8.7%)	1,372 (15%)	10,535 (35%)	3,803 (24%)
Hypertension	30,064 (45%)	4,921 (54%)	17,697 (59%)	9,973 (63%)
Brain Injury	540 (0.8%)	90 (1.0%)	265 (0.9%)	168 (1.1%)
**SDOH**				
Neighborhood Physical and Social Disorder	−0.25 (0.81)	−0.27 (0.89)	−0.19 (0.90)	−0.14 (0.91)
Social Support	−0.16 (0.98)	−0.43 (0.89)	−0.90 (0.90)	−0.01 (0.89)
Social Cohesion	−0.21 (0.94)	−0.09 (0.91)	−0.19 (0.92)	−0.13 (0.92)
Cohen Perceived Stress	−0.18 (0.95)	−0.61 (0.84)	−0.80 (0.89)	−0.42 (0.92)
Loneliness	−0.24 (0.86)	−0.69 (0.74)	−0.72 (0.78)	−0.39 (0.83)
Everyday Discrimination	−0.23 (0.72)	−0.67 (1.14)	−0.28 (0.82)	−0.13 (1.17)
Discrimination in Health Care Settings	−0.18 (0.85)	−0.09 (1.13)	−0.21 (0.90)	−0.09 (1.04)
Walking and Bicycling	0.03 (1.01)	0.14 (1.02)	0.09 (1.00)	0.01 (0.98)
Daily Spiritual Experiences	0.04 (1.01)	0.27 (0.99)	0.10 (1.00)	0.05 (0.95)
Crime Safety	−0.26 (0.71)	−0.53 (0.89)	−0.26 (0.79)	−0.15 (0.82)
Housing Instability	−0.06 (0.81)	−0.05 (1.02)	−0.06 (0.76)	−0.02 (0.82)
Bad Housing Quality	7,197 (12%)	2,374 (27%)	3,449 (12%)	7,047 (46%)
Food Insecurity	3,297 (5.0%)	1,485 (16%)	2,370 (7.9%)	6,998 (44%)

**Table A. 2:** Constructs and measures in the AllofUs SDOH survey.

Construct	Measure
**Social and community context**	
Social cohesion among neighbors	Social cohesion neighborhood scale (4-item)
Social support	RAND MOS social support survey (8-item)
Loneliness	UCLA loneliness scale (8-item)
Perceived discrimination	Everyday discrimination scale (9-item), plus attribution (1-item)
Perceived stress	Cohen’s perceived stress scale (10-item)
Daily spiritual experiences	Fetzer multidimensional measure of religiousness/spirituality (6-item)
**Economic stability**	
Food insecurity	Children’s Health Watch Hunger vital sign (2-item)
Housing instability indicator	Health begins upstream risk screening (1-item)
Housing quality	Accountable health communities (1-item)
**Neighborhood and built environment**	
Neighborhood physical&social disorder	Ross–Mirowsky perceived neighborhood disorder scale (13-item physical disorder subscale)
Neighborhood walkability	Physical activity neighborhood environment scale (PANES) (5 core items)
Neighborhood crime	Physical activity neighborhood environment scale (PANES) crime (2-item subscale)
**Health and healthcare**	
Perceived discrimination in medical care settings	Everyday discrimination scale in medical settings (7-item)

**Table A. 3:** Medication classes and included agents

Medication Class	Included Agents
**Antihypertensives**	Beta-blockers; diuretics; ACE inhibitors; ARBs; calcium channel blockers; alpha blockers (doxazosin, prazosin, terazosin); vasodilators (minoxidil, hydralazine); central alpha-2 receptor agonists (methyldopa, guanfacine, clonidine)
**Non-GLP Antidiabetics**	Alpha-glucosidase inhibitors; biguanides; bromocriptine; DPP-4 inhibitors; meglitinides (nateglinide, repaglinide); SGLT2 inhibitors; sulfonylureas; metformin;
**GLP class**	GLP-1 receptor agonists (Semaglutide, dulaglutide, liraglutide)

**Table A. 4:** Latent construct loadings on social determinants, lifestyle, and comorbidity measures.

Measure	Modality	LC1	LC2	LC3
Housing Quality	SDOH	0.05	0.12	1.53
Food Insecurity	SDOH	0.13	−0.03	2.05
Current Smoker	Lifestyle	−0.27	−0.21	0.12
Alcohol Misuse	Lifestyle	−0.07	0.06	0.04
Stroke	Comorbidity	0	−1.56	0.09
Type 2 Diabetes	Comorbidity	0.13	−1.79	0.44
Heart Failure	Comorbidity	0.12	−1.86	0.31
Hearing_Loss	Comorbidity	−0.14	−1.07	−0.20
Depression	Comorbidity	−0.25	−1.14	0.84
Hypertension	Comorbidity	−0.14	−3.17	−0.15
Brain Injury	Comorbidity	−0.03	−1.37	0.27
Social Cohesion	SDOH	0.19	0.15	0.73
Neighborhood Physical and Social Disorder	SDOH	0.65	0.42	0.53
Walking and Bicycling	SDOH	−0.26	−0.22	0.17
Crime Safety	SDOH	1.39	0.85	0.05
Loneliness	SDOH	−0.26	−0.09	1.23
Everyday Discrimination	SDOH	−0.18	0.02	1.20
Discrimination in Health Care Settings	SDOH	−0.20	−0.02	1.00
Housing Instability	SDOH	0.02	0.03	0.24
Cohen Perceived Stress	SDOH	−0.20	0.02	1.06
Daily Spiritual Experiences	SDOH	−0.23	−0.05	0.22
Social Support	SDOH	−0.14	−0.05	0.80

**Table A. 5:** Baseline characteristics of participants by development of MCI during follow-up

Characteristic	Overall N = 121,322^1^	No MCI N = 120,736^1^	MCI N = 586^1^	p-value^2^
Cluster				<0.001
I	66,422 (55%)	66,231 (55%)	191 (33%)	
II	9,039 (7.5%)	9,009 (7.5%)	30 (5.1%)	
III	30,070 (25%)	29,783 (25%)	287 (49%)	
IV	15,791 (13%)	15,713 (13%)	78 (13%)	
Age	0.17 (0.95)	0.16 (0.95)	0.74 (0.94)	<0.001
Gender				<0.001
Female	74,270 (61%)	73,974 (61%)	296 (51%)	
Male	47,052 (39%)	46,762 (39%)	290 (49%)	
Race				0.028
Non-White	15,914 (13%)	15,855 (13%)	59 (10%)	
White	105,408 (87%)	104,881 (87%)	527 (90%)	
Ethnicity				0.089
Non-Hispanic	110,172 (91%)	109,628 (91%)	544 (93%)	
Hispanic	11,150 (9.2%)	11,108 (9.2%)	42 (7.2%)	
Education attainment				>0.9
Below college	46,794 (39%)	46,568 (39%)	226 (39%)	
College & above	74,528 (61%)	74,168 (61%)	360 (61%)	
Antihypertensives user	40,673 (34%)	40,270 (33%)	403 (69%)	<0.001
Non-GLP Antidiabetics user	13,320 (11%)	13,211 (11%)	109 (19%)	<0.001
GLP Class user	4,657 (3.8%)	4,613 (3.8%)	44 (7.5%)	<0.001

1 n (%); Mean (SD)

2 Pearson's Chi-squared test; Wilcoxon rank sum test
